# SRM 2460/2461 Standard Bullets and Casings Project

**DOI:** 10.6028/jres.109.040

**Published:** 2004-12-01

**Authors:** J. Song, E. Whitenton, D. Kelley, R. Clary, L. Ma, S. Ballou, M. Ols

**Affiliations:** National Institute of Standards and Technology, Gaithersburg, MD 20899; Bureau of Alcohol, Tobacco and Firearms (ATF), Ammendale, MD 20705

**Keywords:** ballistics, bullet signature, standard reference material, SRM, standard bullet, standard casing

## Abstract

The National Institute of Standards and Technology Standard Reference Material (SRM) 2460/2461 standard bullets and casings project will provide support to firearms examiners and to the National Integrated Ballistics Information Network (NIBIN) in the United States. The SRM bullet is designed as both a virtual and a physical bullet profile signature standard. The virtual standard is a set of six digitized bullet profile signatures originally traced from six master bullets fired at the Bureau of Alcohol, Tobacco and Firearms (ATF) and the Federal Bureau of Investigation (FBI). By using the virtual signature standard to control the tool path on a numerically controlled diamond turning machine, 40 SRM bullets were produced. A profile signature measurement system was established for the SRM bullets. The profile signature differences are quantified by the maximum of the cross correlation function and by the signature difference between pairs of compared profile signatures measured on different SRM bullets. Initial measurement results showed high reproducibility for both the measurement system and production process of the SRM bullets. A traceability scheme has been proposed to establish the measurement traceability for nationwide bullet signature measurements to NIST, ATF and FBI. Prototype SRM casings have also been developed.

## 1. Introduction

During the sniper shootings in the Washington D.C. area in October 2002, it became clear that some of the key evidence linking the shootings were the bullet fragments recovered from the crime scenes. This drew attention to the science and technology of ballistics identification, and to a system known as the National Integrated Ballistics Information Network (NIBIN), in which images of bullets and casings collected from crime scenes are matched against a database of previously recorded images [1].

Bullets and casings when fired or ejected from guns pick up characteristic signatures, which are unique to the weapon. Striations on the bullet are caused by its passage through the gun barrel. Marks on the casing are caused by impact with the firing pin, breech face and ejector. By analyzing these signatures, firearm examiners can connect a firearm to criminal acts. In the early 1990s, the IBIS^2^ (Integrated Ballistics Identification System) and the DRUGFIRE system were established for this purpose in laboratories of the Bureau of Alcohol, Tobacco and Firearms (ATF) and the Federal Bureau of Investigation (FBI), respectively [1–3]. Both systems were based on image capture, image analysis and database techniques. In 1998, ATF and FBI initiated a joint project to establish the National Integrated Ballistics Information Network (NIBIN) [1–3]. In December 2002, after a nearly 2 year effort, computer specialists finished installing IBIS workstations into the last of the 233 U.S. crime labs slated to be on the NIBIN [1].

The IBIS is a computerized bullet and casing signature comparison system. It consists of a data acquisition station and a signature analysis station. The data acquisition system uses an optical microscope which captures an image of the bullet or casing surface in a camera. The correlation algorithm of the signature analysis station produces scores based on the degree of similarity between the reference image and each specimen, thus providing a list of high confidence candidates sorted in either ascending or descending order. For bullet image signature comparisons, the IBIS score may be calculated between a single pair of land images or between two sets of images containing, say, six lands each. In order to ensure that both the optical-imaging station and the image-analyzing station of the ballistics identification systems work correctly, bullets are fired and collected at both the ATF National Laboratory Center and the FBI Central Laboratory under a standardized firing procedure. The bullets are intended for measurement quality control of IBIS systems and are called QA (Quality Assurance) bullets. However, it has been found that the bullet signatures of the QA bullets, even fired from the same gun, showed significant differences, and that the signatures change over time. In December 1995, ATF firearm examiner R. Thompson wrote to the Director of the Office of Law Enforcement Standards (OLES) at the National Institute of Standards and Technology, outlining the concept of mass-producing quality assurance standards for bullets and casings. These bullets and casings would bear such a high degree of reproducible and recognized patterns of bullet signatures, that they could be tested at different IBIS locations, which would then maintain scores within a relative consistency and rank.

In response, through OLES funding obtained from the National Institute of Justice (NIJ), the Precision Engineering Division at NIST started a project on SRM (Standard Reference Material) 2460/2461 standard bullets and casings. In the following sections, we discuss the design and manufacture of the SRM bullets in Sec. 2, introduce a bullet profile signature measurement system and initial measurement results in Sec. 3, and pro pose a traceability system for nationwide ballistics measurements in Sec. 4. The prototype SRM casings and initial test results are described in Sec. 5.

## 2. A Virtual/Physical Bullet Profile Signature Standard

### 2.1 Basic Technical Requirements

According to the VIM (International Vocabulary of Basic and General Terms in Metrology) [4], a reference material is defined as:

material or substance one or more of whose property values are sufficiently homogeneous and well established to be used for the calibration of an apparatus, the assessment of a measurement method, or for assigning values to materials.

With this definition in mind, we have developed technical requirements for the standard bullets [5]:

- Real bullet surface characteristics: As a reference standard for the ballistics identification system, the bullet signature must be obtained from real bullets, and duplicated on the surface of the physical standards, the SRM bullets, with high fidelity. The shape, size, material and color of the physical standard bullets should be as close as possible to those of real bullets.
- Repeatability and reproducibility: As a measurement standard, the profile signatures on the SRM bullets must show such a high degree of repeatability and reproducibility that when these SRM bullets are distributed for checking instrument calibrations, they all play the same function as a single SRM bullet. Repeatability here means that the profile signatures are essentially identical over different SRM bullets produced at the same time. Reproducibility means that the profile signatures are highly uniform among different SRM bullets produced over time.
- Measurement traceability: The profile signature measurements for SRM bullets performed at NIST Surface Calibration Laboratory must be traceable to the SI unit of length. By using these SRM bullets, bullet image signature measurements using optical techniques performed at local forensic laboratories can be traced to the ATF National Laboratory Center and to the FBI Central Laboratory.
- Information technology: It is advantageous to base the SRM bullets on information technology. Then the digitized profile signatures may be stored in a NIST computer, and used to produce and re-produce the identical SRM bullets at anytime.

Considering the above technical requirements, the SRM 2460 standard bullet is designed as both a virtual and a physical bullet signature standard described in the following.

### 2.2 Establishment of a Virtual Bullet Signature Standard

The SRM bullets are designed with six land impressions. The virtual standard of the bullet profile signatures is a set of six digitized profile signatures stored in a NIST computer. In 2000, the ATF National Laboratory Center and the FBI Central Laboratory provided NIST with a total of six master bullets, three from each agency. They were fired from different guns under standardized firing conditions. These master bullets underwent profile measurements at the NIST Surface Calibration Laboratory using a commercial stylus instrument. Each bullet was traced on one land. The resulting set of six digitized bullet profile signatures was stored in a NIST computer as the virtual standard for both the fabrication and for the control of measurements of the SRM bullets [5].

### 2.3 Manufacture of the Physical Standard—SRM 2460 Standard Bullets

The Precision Engineering Division at NIST has a long history of developing surface roughness specimens [6–9]. In 1998, a virtual/physical random profile surface roughness standard was developed at NIST using a numerically controlled (NC) diamond turning process [9]. Based on the same NC diamond turning technique for manufacturing random profile roughness specimens, two prototype standard bullets were developed in April 1998 [10,11]. From 1998 to 2001, tests were conducted on these prototype standard bullets. The results showed high uniformity and reproducibility for profile signatures [10,11]. Comments on them were collected from firearm examiners at ATF and FBI. From this information, improvements were made to both the design and manufacturing process for SRM 2460 standard bullets. In January 2002, 20 SRM bullets were produced and in June 2003, another 20 bullets were produced.

**Fig. 1:**
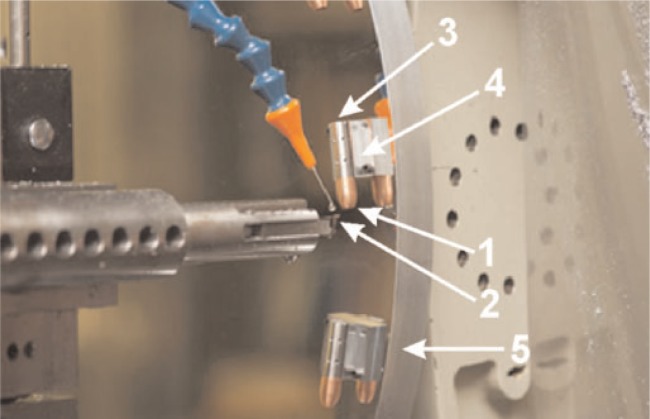
Manufacturing setup for SRM 2460 standard bullets: One of twenty SRM bullets (1) was cut by a diamond tool (2). Each bullet was fastened to a cylinder (3), which was mounted on a “V” block (4) set on an aluminum wheel (5). 20 SRM bullets were manufactured at the same time.

**Fig. 2:**
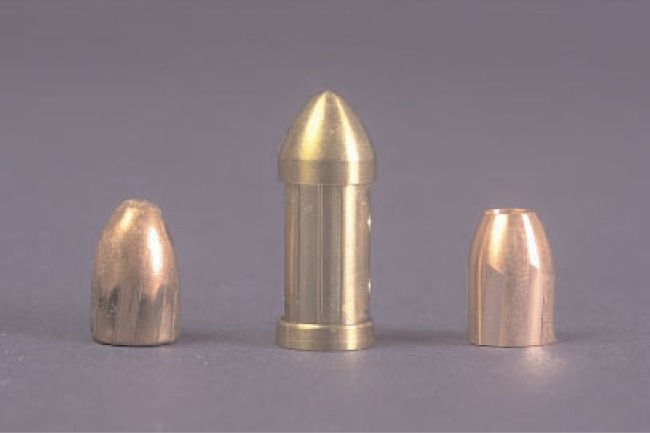
A master bullet from ATF National Laboratory Center (left), a prototype standard bullet (center), and a SRM 2460 standard bullet (right).

Because the SRM bullets are manufactured by NC diamond turning the profile signature onto the bullet surface, bright acid copper is the preferred material for electroforming the substrate. The deposit is electroplated onto copper cylinders, which are then used for the diamond turning process [12]. Figure 1 shows the manufacturing setup on a numerically controlled diamond turning machine at the NIST Instrument Shop. The set of standard bullets (1) were cut by a diamond tool (2). The bullets were fastened to cylinders (3), which were mounted on “V” blocks (4) set on an aluminum wheel (5). Twenty SRM bullets were manufactured at the same time. After cutting one signature, the cylinders (3) were rotated 60° to cut the next signature until all six signatures were completed. There was a 5° tilt for the “V” block (4) alignment on the aluminum wheel (5), so that a 5° twist in the bullet signature land could be made on the SRM bullets. That gave the SRM bullet a shape close to that of the master. Detailed information for the manufacturing process and quality control can be found in Ref. [12]. Figure 2 shows a SRM 2460 standard bullet (right) with a master bullet from the ATF National Laboratory Center (left), and a prototype standard bullet (center). The duplicated bullet signatures on the surface of the standard bullets are highly two-dimensional, and hence are very smooth along the machining direction perpendicular to the bullet signature. Therefore, these bullets may be characterized by their topographic profile signatures, machined on the land engraved areas and measured, for example, with a stylus instrument.

## 3. A Bullet Profile Signature Measurement System

Measurement results showed that the bullet profile signatures on the standard bullets can be manufactured with high uniformity [10,11]. However, there were small differences when comparing profile signatures of the SRM bullets with the virtual bullet signature standard, and when comparing the signatures between different SRM bullets. It is therefore important to define a traceable parameter and to develop a measurement system for measuring profile signature differences. This parameter is particularly important for the production, inspection and quality control of the SRM bullets.

It was decided to adapt the signal processing functions of auto-correlation and cross-correlation for the bullet signature comparisons [13,14]. Because the bullet signatures can be considered as random profiles, their auto-correlation functions (ACF) decay with increase in the absolute value of the shift distance. This statistical property is very useful for the bullet profile signature comparisons for quantifying the difference of bullet signatures. When two bullet profile signatures are compared with each other, one is taken as the reference signature A and the other is the compared signature B:

- If the two profile signatures are exactly the same, A = B, their ACF achieves the maximum value (1.00) when the shift distance is zero.
- If two compared profile signatures A and B have essentially the same pattern but small differences in the profile details, then A ≅ B. For example, when two bullets are fired from the same gun, their profile signatures may have strong correlation. With the shift distance changing, their CCF will have a maximum value but not as large as 1.00, because there are some differences between these two correlated profile signatures.
- If signatures A and B come from bullets fired from different guns, there should be only small correlation between the two bullet signatures, B ≠ A. With the shift distance changing, only random variations appear on the CCF curve without a clear correlation peak.

From the cross-correlation function (CCF) we calculate a parameter called *CCF*_max_, which is the maximum value of the CCF when the two compared profiles are in phase. Although the *CCF*_max_ can be used for the profile signature comparison, it is not a unique parameter. Based on the definition of the cross-correlation function [13], if two compared signatures have the same shape but different vertical scales, their *CCF*_max_ is still 100 % even if they are two different signatures. Therefore, a parameter, signature difference *D*_s_, is proposed for quantifying the profile signature differences [13]. It is calculated as follows:

- At the shift where the *CCF*_max_ between signature B and A occurs, construct a new profile *Z*_B − A_ (X), which is equal to the difference of the compared profile signature *Z*_B_ (X) and the reference profile signature *Z*_A_ (X):
  $$Z_{B-A}\;(X)=Z_{B}\;(X)-Z_{A}\;(X).$$ (1)
- Calculate the *Rq* (root-mean-square roughness, see Ref. [15]) value for the new profile *Z*_B − A_ (X). We call this parameter *Rq*(B − A).
- Calculate the signature difference *D*_s_ between signatures B and A defined as
  $$D_{s}=Rq^{2}(B-A)/Rq^{2}(A)$$ (2)

where *Rq*^2^(A) is the mean square roughness of the reference signature *Z*_A_ (X), used here as a comparison reference. From Eqs. (1) and (2), it can be seen that when two compared profile signatures are exactly the same, *Z*_B − A_ (X) = 0, then *Rq*^2^(B − A) = 0, and *D*_s_ = 0.

**Fig. 3:**
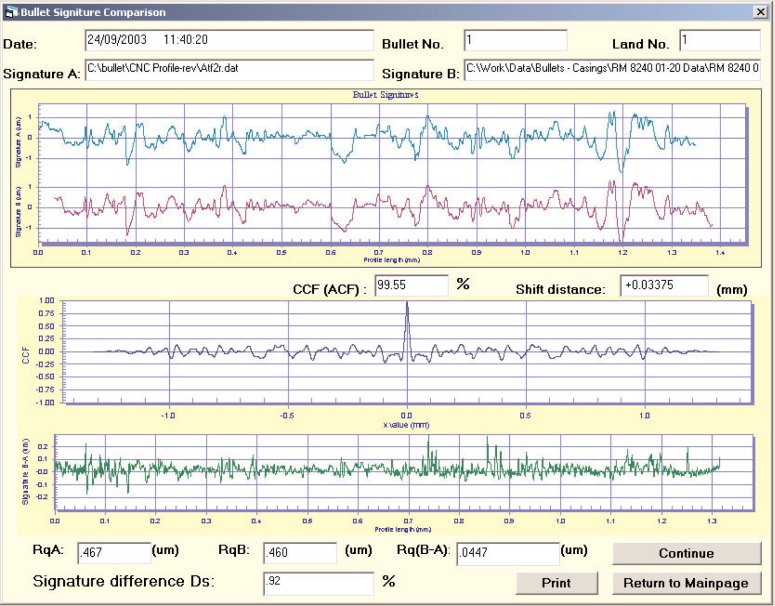
Bullet signature comparison between the signature of the No. 1 land of S/N 001 SRM 2460 (Signature B, the second profile from the top) and the virtual standard (Signature A, the top profile). The maximum of the cross correlation function *CCF*_max_ = 99.55 %, the signature difference *D*_s_ = 0.92 %. The profile difference of two signatures, B − A, is shown as the bottom profile with different scale.

A bullet profile signature measurement program was developed to calculate the above parameters. A screen output of the measurement program is shown in Fig. 3. These modified profiles result after curvature removal, resampling, and bandpass filtering (2.5 μm to 250 μm) of the measured profiles [12,14]. The top profile is the virtual profile signature standard, or “Signature A”, which is one of the six modified profiles from the bullet signatures of the six master bullets provided by the ATF and the FBI. The second profile shows the measured bullet signature, or “Signature B”, which in this case is a profile signature from the No. 1 land impression of the S/N SRM 2460-001 bullet. Both profiles have a data spacing of 0.25 μm. The cross correlation peak *CCF*_max_ is equal to 99.55 %. At this position, a new profile, Signature (B − A) (see the bottom profile in Fig. 3), is constructed, which is equal to the difference between the two compared signature profiles. Then the signature difference *D*_s_ is calculated from Eq. (2) to be 0.92 %.

**Fig. 4:**
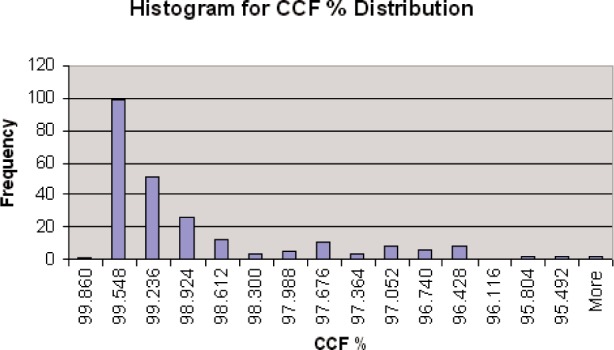
*CCF* distribution for 240 bullet signatures of 40 SRM bullets. All *CCF* values are higher than 95 %, most are even higher than 99 %. *CCF* = 100 % means the measured bullet signature is exactly the same as the virtual bullet signature standard (point by point).

The six signatures on each of the 40 SRM bullets have been measured. The measurement results show that all *CCF*_max_ values for the 240 measured profile signatures, when compared with the virtual profile signature standard, are higher than 95 %, and most are higher than 99 % (Fig. 4). Considering that the measured profile signature is exactly the same as the virtual standard when *CCF*_max_ = 100 % and *D*_s_ = 0, the measurement results have demonstrated high reproducibility for both the NIST bullet signature measurement system and the SRM bullets. Measurement results also showed that the machined profiles are highly two-dimensional and therefore are very smooth along the machine direction. In other words, they are highly uniform and yield the same profile along the land impression of the SRM bullets. Based on NIST Technical Note 1297 [16], an uncertainty analysis procedure is currently in progress to report the measurement results for both *CCF*_max_ and *D*_s_ with a 95 % confidence level.

Compared with existing commercial algorithms for bullet and casing image signature comparisons, The proposed *CCF*_max_ and *D*_s_ parameters have several features [13,14]:

- They are easy to understand and use, and are traceable to the length standard and the virtual bullet signature standard.
- The same basic parameter and algorithm can be used for quantifying signature differences for both 2D-profile bullet signatures and 3D-topography signatures. A prototype 3D-topography characterizing system for standard cartridge casings has been developed recently.
- From Eqs. (1) and (2), it can be seen that for the collection of all 2D-profile and 3D-topography comparisons, the minimum signature difference is *D*_s_ = 0, which occurs when, and only when, these two profiles or topographies are exactly the same. That means, when any two compared 2D-profiles or 3D-topographies have a signature difference of *D*_s_ = 0, these two profiles or topographies must be exactly the same (point by point).
- The measurement results of 240 bullet signatures on 40 SRM 2460 standard bullets showed that there is a strong linear correlation between *CCF*_max_ and *D*_s_ [14]. Therefore, in practice, either parameter can be used for representing bullet signature differences of the SRM bullets.
- The characteristic parameters for bullet and casing signature comparisons, *CCF*_max_ and *D*_s_, are directly derived from a statistical process, and have a potential for ballistics comparisons of bullet and casing signatures. One drawback of this approach is that scale changes and nonlinearities of the instrument in the *X*- and *Y*-directions could detract from the observation of high correlation between similar profiles or topography images. However, for a well-calibrated bullet and casing signature measurement system, this effect should be very small.

## 4. Traceability for Nationwide Bullet Signature Measurements

The profile signature measurements for SRM bullets must be traceable to the SI unit of length. This is ensured by using a set of calibration and check standards, and standardized measurement and uncertainty procedures in the NIST Surface Calibration Laboratory [17].

**Fig. 5:**
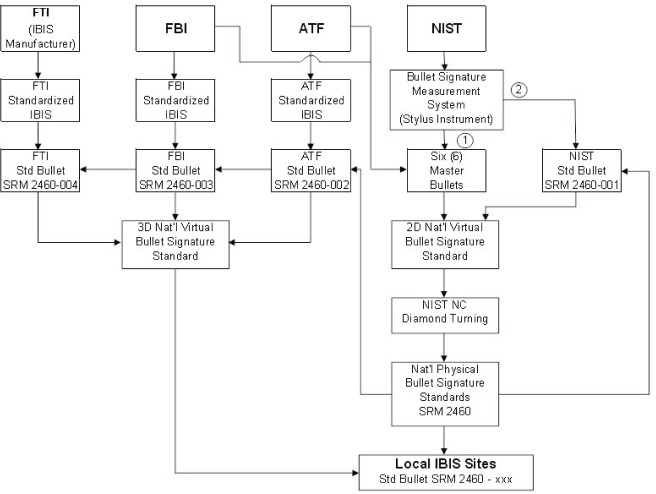
A proposed national bullet signature measurement traceability and standardization system. The arrows indicate different types of functions such as measurement, calculation and simulation.

In order to establish measurement traceability of image signature measurements at local laboratories to NIST, ATF and FBI, we propose to use SRM 2460 standard bullets following a flow diagram shown in Fig. 5 starting with the six master bullets that ATF and FBI provided. The six master bullets were profiled at the NIST Surface Calibration Laboratory using a stylus instrument, as shown in Fig. 5, branch 1. The set of six digitized 2D profile signatures was used as a 2D virtual standard for profile signatures to control a numerically controlled diamond turning machine at NIST to produce the SRM bullets as physical standards. One of them, numbered SRM 2460-001, would be kept at NIST as a check standard. This check standard would be routinely measured at the NIST Surface Calibration Laboratory, and compared with the virtual standard for measurement quality control, as shown in Fig. 5, branch 2. Other SRM bullets would be distributed to three ATF Laboratory Centers, to the FBI Central laboratory, and to FTI (Forensic Technology Inc., Montreal, Canada, IBIS manufacturer), leaving the remaining SRM bullets for purchase through the NIST Standard Reference Material (SRM) Office with the intent that these bullets would be utilized by firearm examiners on their respective IBIS. At the ATF National Laboratory Center, the SRM bullets would be measured under a set of pre-determined standardized IBIS testing conditions. The resulting digitized optical intensity images would be used as the image signature standard and be transferred to local IBIS sites. In principle, a virtual standard for the 3D image signatures might also be generated by computer-imaging techniques using the 2D virtual profile signature standard as input, and might be verified by measured IBIS images on SRM bullets. By measuring these SRM bullets at local IBIS sites, and comparing the measured images with those of the image signature standard measured at ATF under standardized conditions, differences in IBIS calibrations and testing conditions between the local IBIS sites and ATF can be detected. That could enable traceability for ballistics measurements nationwide to NIST, ATF, and FBI, and ensure the measurement quality control of local IBIS sites [18].

## 5. SRM 2461 Standard Casings

Electroforming is a commercial technique that has been successfully used for replication of surface roughness specimens [8]. The same technique is being utilized for the manufacture of SRM 2461 standard casings. In April 1998, five master casings were produced by a standardized firing procedure at the ATF National Laboratory Center. By replication of two master casings, 21 prototype standard casings were manufactured by two manufacturers [19]. The IBIS was used for the initial tests of these prototype standard casings. For casing signature comparisons, the IBIS correlation score may be calculated between a pair of images of either the firing pin impression, the ejector mark or the breech face impression.

**Fig. 6:**
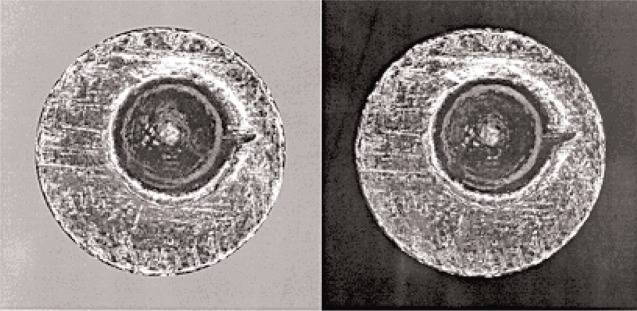
Firing pin and breech face image comparison between P11 (left) and P21 (right) prototype standard casings.

**Fig. 7:**
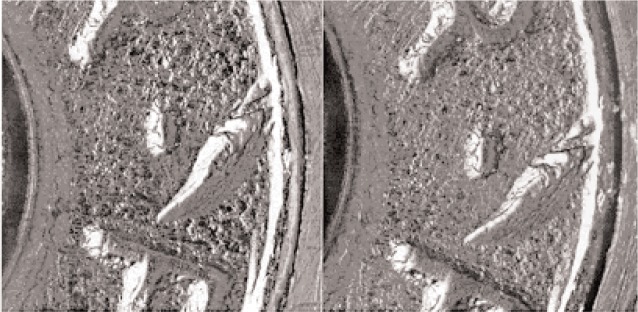
Ejector image comparison between P21 (left) and P31 (right) prototype standard casings.

From the image comparisons between different prototype standard casings, it can be seen that these images show high reproducibility. For example, Fig. 6 shows firing pin and breech face signature comparisons between No. P11 and No. P21 prototype standard casings. Figure 7 compares ejector mark impressions between the No. P21 and No. P31 casings. Both figures show close agreement between the two compared casing images. The topography signature comparisons between the firing pin of No. P21 and P31 casings have also been conducted using the recently developed casing topography signature comparison system, which is a 3D version of the bullet profile signature comparison system, as mentioned before. The preliminary comparison result shows *CCF*_max_ = 99.09 %. These comparisons indicate good surface reproducibility between different prototype standard casings. Based on these initial test results, the basic design for the SRM 2461 standard casings has been made, and the SRM casings are planned for fabrication in the near future.

## 6. Summary

A new standard for ballistics measurements—SRM 2460/2461 standard bullets and casings—is being developed at NIST. The SRM 2460 standard bullet is designed as both a virtual and a physical standard. The virtual standard is a set of six digitized bullet profile signatures acquired from six master bullets provided by ATF and FBI. From these profile signatures, 40 physical standards, the SRM bullets were produced in the NIST Instrument Shop.

A new parameter, signature difference *D*_s_, was developed for both the 2D bullet profile signature and 3D casing topography signature comparisons using auto- and cross-correlation functions. A bullet signature measurement system has been established at NIST for the measurements of the standard bullets and standard casings. Measurement results for 40 SRM bullets with a total of 240 signatures show very high reproducibility. A traceability system is proposed to establish nationwide ballistics measurement traceability to NIST, ATF, and FBI. Prototype standard casings have also been developed at NIST using an electro-forming technique. SRM 2461 standard casings are planned for fabrication.

The SRM bullets and casings aim to support the National Integrated Ballistics Information Network (NIBIN) in the United States. They can be used for checking instrument calibration, for maintaining quality control in ballistics measurements, and for promoting a nationwide ballistics measurement traceability system, and therefore, have great potential to support nationwide crime fighting and to strengthen homeland security.

## Footnotes

2 Certain commercial equipment, instruments, or materials are identified in this paper to foster understanding. Such identification does not imply recommendation or endorsement by NIST, nor does it imply that the materials or equipment identified are necessarily the best available for the purpose.
